# The ethics of community water fluoridation: Part 1 - an overview of public health ethics

**DOI:** 10.1038/s41415-024-8058-4

**Published:** 2025-03-14

**Authors:** Bhavini Patel, Alison Patrick, Thomas Anthony Dyer

**Affiliations:** 41415286263001 Dental Practitioner and Alumnus, School of Clinical Dentistry, University of Sheffield, 19 Claremont Crescent, Sheffield, S10 2TA, UK; 41415286263002 Senior University Teacher in Law and Ethics, School of Clinical Dentistry, University of Sheffield, 19 Claremont Crescent, Sheffield, S10 2TA, UK; 41415286263003 Senior Clinical Teacher, School of Clinical Dentistry, University of Sheffield, 19 Claremont Crescent, Sheffield, S10 2TA, UK

## Abstract

**Aim** To provide an overview of public health ethics, how it differs from medical ethics, and why this is important when considering the justification of public health interventions, such as community water fluoridation.

**Method** Narrative review of the literature.

**Results** Like medical ethics, public health ethics is underpinned by moral-based theories: consequentialism and non-consequentialism. Utilitarianism is an example of the former and sees moral action as that which produces the overall greatest benefit or wellbeing in society. In contrast, non-consequentialist theories focus on whether an action is right or wrong regardless of consequences. One such approach is principlism, where respect for autonomy, beneficence (benefit), non-maleficence (avoidance of harm), and justice are considered. However, as most public health interventions restrict autonomy to some extent, these require modification to balance this with any collective benefit. Similarly, political theory influences public health ethical thinking: liberalism's focus on autonomy and avoidance of infringement of freedoms challenges many public health interventions. Given these complexities, frameworks exist to help guide ethical deliberation in public health.

**Conclusion** Various principles and ethical frameworks have been proposed for public health interventions. They have more utility when considering the ethics of interventions, such as community water fluoridation, than those for medical interventions.

## Key points

- This paper is the first of two that consider the ethics of community water fluoridation. It reviews public health ethics and how it differs from medical ethics, describing its underpinning moral and political theories and how these lead to different perspectives.
- Understanding and considering such perspectives is essential. For example, acknowledging and addressing tensions between the rights of individuals and population health benefit.
- Proposed principles and frameworks are outlined that aim to balance different perspectives in ethical deliberations for public health interventions, such as community water fluoridation.
- References to policy centre on the United Kingdom; consequently, relevance to other countries will vary.

## Introduction

Community water fluoridation (abbreviated to ‘fluoridation' in this report) has been defined as the regulated addition of a fluoride compound to public drinking water with the aim of improving oral health and wellbeing of populations by reducing caries experience and inequalities.^1^^,^^2^ In the 1930s, evidence emerged that dental caries was inversely correlated with naturally occurring fluoride in water, which led to the introduction of fluoride to public supplies in the United States (US) in the mid-1940s and in the following decades in the United Kingdom (UK).^3^ Globally, approximately 400 million people in 25 countries now receive fluoridated water, with many schemes operating for over 70 years.^4^^,^^5^ An additional 50 million receive water with naturally occurring fluoride at around the same concentration used in fluoridation schemes.^6^

However, debate on fluoridation's adverse effects on oral and general health increased in the early 1970s. In addition to dental fluorosis, there were concerns it may have other negative health impacts and has been linked to a range of health conditions. This fuelled ongoing debates on the justification for fluoridation. Supporters argue it is an effective measure which benefits people regardless of socioeconomic position or access to services and its benefits outweigh any harms. Others emphasise safety concerns and putative harmful effects and cite an individual's right to choose what is added to public water supplies.^3^^,^^7^ Consequently, fluoridation is socially and politically controversial and its justification continues to be debated. Historically, similar concerns have been raised about vaccination programmes, seatbelt legislation, smoking bans in enclosed public places, and social distancing in the COVID-19 pandemic.

A key consideration when justifying the introduction and maintenance of any public health intervention is its ethics.^8^ This paper aims to explore public health ethics. In doing so, it considers how it differs from medical ethics, the moral and political philosophies that underpin it, how these influence perspectives, and how it can be assessed. Where appropriate, fluoridation is used to illustrate descriptions and arguments made.

Public health interventions commonly generate tension between the rights of individuals and the benefits for communities and society as a whole, which raises different ethical concerns compared to medical interventions.^9^ Simply applying medical ethics to public health interventions would risk prioritising individual rights over collective benefit, and therefore a more nuanced approach is required.

Like medical ethics, public health ethics is underpinned by moral philosophical theories. Before going further, it is worth differentiating morals and ethics. They are closely related concepts and colloquially, the terms are often used interchangeably. Indeed, they have similar etymology (morals is from the Latin *mos or mores,* ethics is from the Greek *ethos*). However, morals refer mainly to guiding principles and ideas for deciding what is right and wrong, and why. They are more self-determined, with influences from family and education to religion. Ethics commonly refer to rules, actions, or behaviours that are usually informed by moral philosophy.^10^ They tend to be more practical and define allowable actions, such as those created by professions, such as medicine and dentistry.^11^ Moral theories pertinent to public health ethics include those that are consequentialist and non-consequentialist. In addition, political theories can also shape public health ethical perspectives, in particular, liberalism and communitarianism. To be able to consider the ethics and justification of public health interventions, such as fluoridation, it is essential that different perspectives are understood and considered.^12^^,^^13^

Both groups of theories and their impact on public health ethical perspectives will be considered in the following sections.

## Moral-based philosophies

Moral-based philosophies comprise two broad groups: consequentialist and non-consequentialist theories.

### Consequentialist theories

Consequentialism holds that ‘the moral value of an action is determined by its consequence'.^12^ Simply put, the action that delivers the best consequences is the moral choice, where ‘action' in this context refers to acts such as public health policymaking and interventions. One problem is that ‘best consequences' is non-specific and can be interpreted differently. Utilitarianism is a consequentialist moral theory in which the moral choice is the one that produces the greatest benefit in terms of utility to the greatest number.^14^ Generally, public health is a utilitarian endeavour as it aims to maximise population utility in terms of health and wellbeing. Sometimes, this is interpreted as the morally correct action is that which provides benefit for the greatest number. However, the focus should be the greatest benefit for the greatest number. It is also an impartial theory in that it asserts that everyone's utility counts equally i.e. no one person or group's utility, including those close to us, should be valued differently.^12^

Therefore, utilitarian arguments can be made to morally justify fluoridation if it maximises oral health (the utility in this case) by reducing caries, even if a small number are adversely affected.^15^ However, it does not equip us well to balance all consequences of fluoridation and its overly simplistic use has been termed ‘naive utilitarianism'.^12^ For example, how do we judge the relative health benefit and utility of reduction of caries in some with an increase in dental fluorosis (which may or may not require intervention) in others?

Rather than solely taking into account the consequences of actions, others have argued that other factors should be considered in moral judgements. Such non-consequentialist theories can also influence perspectives on public health ethics.

### Non-consequentialist theories

In non-consequentialist theories, moral acts are independent of consequences. Those that relate to what we ought to do regardless of the consequences are referred to as deontology. Virtue theories and principlism are also regarded as non-consequentialist and will also be outlined in this section.

#### Deontological theories

In these theories, duty, obligations and rules are emphasised. These theories shift the focus from a utilitarian view to consider whether it is a morally right action *prima facie.*^15^ In this case, duty and obligations to all individuals should be considered. Immanuel Kant is the best-known deontological theorist who held that we should act in ways that can be universalised and that do not treat persons as a means to an end.^12^ Consequently, in a public health intervention such as fluoridation, this would include consideration of the minority who may be harmed. On the other hand, duty towards vulnerable groups with limited influence should be considered too, for example, children and those without full mental capacity. Depriving such groups of the potential benefits of fluoridation and not meeting their needs may not be a moral act from a deontological perspective.

#### Virtue theories

With roots in ancient and eastern philosophy, virtues are character traits to behave in certain ways, such as kindness, courage and honesty.^12^ The emphasis is on what it is to live and practise well, the outlooks and instinctual reactions we want to promote, and the sort of people we want to make decisions.^9^ In practice, the implications in ethical decision-making are similar to that of deontological theories. For example, when applying this to fluoridation, if the argument of the right to choose what is added to water is not considered, this would be non-virtuous. Yet, if the needs of those at high risk of caries and its sequelae are not considered, this too would be non-virtuous. It has been argued that applying virtue ethics in public health interventions can be seen as ‘practically wise' as it requires the sensitive balancing of the claims of the individual and those of the community, rather than one at the expense of another.^12^ However, critics of virtue ethics point to a lack of a framework to decide what is and what is not virtuous in any given situation.

#### Principlism

Although consequentialist and non-consequentialist theories are helpful in understanding different perspectives, they are of less practical use given the complex considerations required in public health.^12^ A different approach is to use a series of ethical principles, informed by moral theories: so-called principlism. While regarded as a non-consequentialist approach, it is conceptually different.

For many practitioners, ethical evaluation involves applying four bioethical principles described by Beauchamp and Childress:^16^ autonomy; non-maleficence; beneficence; and justice. Although intended for individual patient care, the four principles have been applied to public health interventions. The authors' intention was that each principle is equally important to consider in ethical deliberations. However, respect of autonomy - i.e. the right of an individual to freely choose how to live their life - is often seen as first among equals, particularly in western cultures,^17^ which can be problematic, as autonomy is usually restricted to some extent by most population-based public health measures,^12^ such as fluoridation. Non-maleficence is the principle to cause no harm. In fluoridation, the rule of ‘double effect' could apply here: an adverse outcome (eg dental fluorosis) can be tolerated as long as it was not intended and the primary aim was to do good.^16^ The third bioethics principle of beneficence is the counterpoint to non-maleficence and is the moral obligation to act for the benefit of others. Finally, the principle of justice is described as ‘a group of norms for distributing benefits, risks, and costs fairly'.^16^ This principle is consistent with notions of social justice^18^^,^^19^ and ameliorating disadvantages, such as inequalities within the distribution of resources, which could be consistent with fluoridation's effects.^20^

Although the principles have been applied to public health ethics, their usefulness has been questioned.^12^ First, as has been identified, individual autonomy is normally restricted in public health interventions and therefore its emphasis is inappropriate and ultimately unhelpful. Second, they were intended to be applied to individual patient interventions and not communities. For example, questions about beneficence, non-maleficence and justice, although seemingly relevant, do not adequately address the key dilemma in public health ethics of the right of the individual balanced with community benefit. To address these concerns, principlism has been adapted by various authors for public health interventions.

#### Public health principlism

At the core of public health principlism is the concept that a common citizenship exists and a community can have shared loyalties and duties to itself, including health.^21^^,^^22^ By protecting the community's health, one protects the individual's health. Public health interventions with communal interest, such as fluoridation, extend traditional medical ethics and point to the need for a uniquely public health perspective to ethical thinking.^22^ Various authors have concurred that Beauchamp and Childress' four bioethics principles are inappropriate for evaluating public health interventions but can be used with modification (Table 1).^13^^,^^22^^,^^23^ Upshur^13^ identified four principles and focused on public health policymaking: Mill's harm principle;^24^ least restrictive or coercive means; reciprocity; and transparency. Childress *et al.*^23^ proposed five principles: effectiveness; proportionality; necessity; least infringement; and public justification. Finally, Klugman^22^ saw different principles applicable to public health interventions. He used ideas of solidarity, efficacy, integrity and dignity to guide ethical reasoning and action at a population level. Although the names of principles differ, there is much overlap in the meaning and the moral underpinnings of each.

**Table 1:** Ethical principles proposed as important in the justification of public health interventions

**Authors**	**Principle**	**Descriptor**
Upshur^13^	‘Harm principle'	Power should only be exercised over individuals against their will to prevent harm to others. Derived from J. S. Mill's harm principle
	Least restrictive or coercive means	More restrictive and coercive means should only be used when less restrictive and coercive means have failed
	Reciprocity	Public health interventions may require sacrifices and lead to costs for individuals or communities. Society should seek to compensate those impacted and facilitate their continued roles
	Transparency	All stakeholders should be involved in decision-making, which should be a clear and accountable process, and as free from political and domination of specific interests as possible
Childress *et al.*^23^	Effectiveness	If infringing moral considerations, there must be evidence that public health will be protected
	Proportionality	Probable health benefits must outweigh adverse effects from infringement of moral considerations, such as autonomy
	Necessity	Not all interventions that are effective and proportionate are necessary. If there are alternatives, the least morally problematic should be chosen
	Least infringement	On meeting the first three principles, infringement of moral considerations should be minimised. For example, if autonomy is infringed, the least restrictive alternative should be sought
	Public justification	Where interventions infringe moral considerations, this should be justified publicly. This should be democratic and transparent to establish accountability^22^and build public trust
Klugman^22^	Solidarity	A utilitarian principle, built on equity (benefits should be shared fairly), community autonomy (community representatives decide), and paternalism (infringement of liberty), in which communities come together to improve health
	Efficacy	Evidence that the intervention should be successful in reaching goals, is scientifically sound, and socially, politically, and culturally feasible
	Integrity	The nature and culture of a community should be preserved and respected. The community should be involved in developing interventions so they are consistent with their values
	Dignity	All in communities are of equal worth, deserve the same moral respect, and should be treated accordingly. Wherever possible, the least restrictive intervention should be chosen

In addition to moral-based philosophies, there are two political philosophies informed by moral theory that are pertinent to public health ethics, and these are considered next.

## Political philosophies

The two main philosophies pertinent to public health ethics are liberalism and communitarianism. Arguably, the former has become increasingly influential in societal changes in the UK and other high-income countries, which poses a challenge to many public health interventions.

### Liberalism

In this sense, liberalism does not refer to its more colloquial use in the US of a socially progressive, left-leaning political ideology; rather, it is the right to self-determination based on the concept of autonomy. It emphasises an individual's right to follow their own conception of good and to pursue their lives according to their own beliefs of worth or value. Consequently, a liberal perspective may challenge public health interventions, even if the intention is for the good of population health.^12^

However, as a utilitarian, Mill challenged the right to choose in all cases. In his ‘harm principle', he argues that if choosing an action results in harm to others, it is morally right for a state to politically intervene to prevent that harm.^24^ Although clear in principle, neither Mill nor liberalism is universally helpful in the practical adjudication of interventions. Harms and benefits of interventions vary depending on the context.

Another liberal objection related to the prevention of autonomy is the infringement of personal freedom, where it is seen as the absence of constraint, interference, or restriction: so-called negative freedom.^12^ However, freedom can be conceptualised positively, where one is free to do something, rather than restrained. In this way, interventions such as fluoridation could provide additional freedoms and quality of life offered by better oral health, so could be consistent with a liberal political perspective.

A recent development within liberalism is the notion of libertarian paternalism, which at first analysis seems more consistent with public health interventions. This involves states influencing (or ‘nudging') without coercion, so individuals can make rational choices in their best interest but informed by public health interventions and messaging.^25^ Although this has utility in those able to choose, it does not in those unable to make decisions for themselves, and it is inconsistent with population approaches, such as fluoridation, that should benefit such groups.

### Communitarianism

In contrast to liberalism, communitarianism emphasises the interconnection of individuals and communities and concentrates on how a person's identity is shaped by communities. There is less emphasis on the importance of individuals and a strong focus on shared responsibility.^12^ Consequently, it challenges liberalism in seeing individuals as separate and removed from each other, as it holds that this comes at the expense of the community. The focus on community means that utilitarian perspectives and endeavours are more consistent with the communitarian political philosophy. Consequently, public health interventions like fluoridation are more easily justified from a communitarian perspective.

Finally, it is worth acknowledging that liberal and communitarian perspectives can be compatible in that a population's best interests will also benefit individuals. In addition, most liberal perspectives accept the harm principle i.e. the need to avoid harm to others when exercising the right to choose.^16^ This is one reason why politicians across the political spectrum can be supportive of fluoridation.

## Public health ethical frameworks

Given the variety and complexity of moral and political theoretical perspectives on public health ethics, ethical frameworks have been proposed to help deliberate the justification of interventions.^23^^,^^26^^,^^27^^,^^28^ These define values and pose questions to guide or ‘frame' decisions,^29^^,^^30^ and broadly use a principlism approach (Table 2). Although they differ in scope and applicability, they can be useful analytical tools to guide discussions,^31^ but should be used with care to avoid them being applied rigidly and simplistically.^1^

**Table 2:** An overview of public health ethical guides/frameworks

**Authors/country**	**Format**	**Factors considered when assessing interventions and programmes**	**Moral and ethical values reflected in framework**
Kass^26^ USA	Six questions	The public health goals of the programme Effectiveness of the programme in achieving its goals Known potential burdens of harms of the programmes Minimisation of burdens and harms and alternative interventions Fair implementation of the programme Burdens and benefits are balanced	Wellbeing, benefits, minimising harms, liberty, justice, autonomy, respect, distributive justice
Childress *et al.*^23^ USA	General moral considerations map	Production of benefits Avoidance, prevention, and removal of harms Maximal balance of benefits over harms Distribution of /communication of benefits and burdens fairly and ensuring public participation Respect of autonomous choices and liberty of action Protection of privacy and confidentiality Maintenance of promises and commitments Disclosure of information, honesty, and truthfulness Building and maintaining trust	Wellbeing, utility, benefits, minimising harms, distributive justice, procedural justice, autonomy, liberty, transparency
Nuffield^27^ UK	Two analytical tools: Stewardship model Intervention ladder	**Stewardship model** Protecting and promoting health Ensuring access Reducing risks of ill health Reducing inequalities **Intervention ladd**er Restricting/eliminating of choice Guidance of choices through incentives and disincentives Guidance of choices through changing policy Enabling choice Providing information Doing nothing or mere monitoring	Wellbeing, benefit, minimising harm, distributive justice, fairness, liberty
Tannahill^28^ UK	Decision-making tool based on evidence, ethics and theory	Evidence Theory Ethical principles	Doing good, minimising harm, respect, empowerment, social responsibility, participation, openness, sustainability, accountability, equity

## Conclusion

Public health aims to benefit populations or subpopulations by preventing disease, promoting health and reducing inequalities. This frequently generates tension between the rights of individuals and the requirement to meet the needs of communities, and means approaches intended to appraise the ethics of individual patient care are inappropriate for public health interventions. Given the different moral and political influences on perspectives, various principles and ethical frameworks have been proposed. These identify factors to be considered when deliberating the justification of public health interventions, such as fluoridation. The second paper in this series^32^ is a scoping review which examines how the ethics of fluoridation has been appraised in the literature.

## Data Availability

The data included in the review are available from the authors on request.
